# An Approach to the Investigation of Thrombocytosis: Differentiating between Essential Thrombocythemia and Secondary Thrombocytosis

**DOI:** 10.1155/2024/3056216

**Published:** 2024-02-12

**Authors:** Ala Almanaseer, Benjamin Chin-Yee, Jenny Ho, Alejandro Lazo-Langner, Laila Schenkel, Pratibha Bhai, Bekim Sadikovic, Ian H. Chin-Yee, Cyrus C. Hsia

**Affiliations:** ^1^Department of Medicine, London Health Sciences Centre, London, Ontario, Canada; ^2^Division of Hematology, Department of Medicine, London Health Sciences Centre, London, Ontario, Canada; ^3^Molecular Diagnostic Division, Department of Pathology and Laboratory Medicine, London Health Sciences Centre, London, Ontario, Canada; ^4^Department of Pathology and Laboratory Medicine, London Health Sciences Centre, London, Ontario, Canada

## Abstract

**Background:**

Thrombocytosis is a common reason for referral to Hematology. Differentiating between secondary causes of thrombocytosis and essential thrombocythemia (ET) is often clinically challenging. A practical diagnostic approach to identify secondary thrombocytosis could reduce overinvestigation such as next generation sequencing (NGS) panel.

**Methods and Results:**

All adult patients with thrombocytosis (≥450 × 10^9^/L) who underwent molecular testing at a single tertiary care centre between January 1, 2018 and May 31, 2021 were evaluated. Clinical and laboratory variables were compared between patients with secondary thrombocytosis vs. ET. Clinical variables included smoking, thrombosis, splenectomy, active malignancy, chronic inflammatory disease, and iron deficiency anemia. Laboratory variables included complete blood count (CBC), ferritin, and myeloid mutations detected by NGS. The overall yield of molecular testing was 52.4%; 92.1% of which were mutations in *JAK2*, *CALR*, and/or *MPL*. Clinical factors predictive of ET included history of arterial thrombosis (*p* < 0.05); active malignancy, chronic inflammatory disease, splenectomy, and iron deficiency were associated with secondary thrombocytosis (*p* < 0.05). A diagnosis of ET was associated with higher hemoglobin, mean corpuscular volume (MCV), red cell distribution width (RDW), and mean platelet volume (MPV), while secondary thrombocytosis was associated with higher body mass index, white blood cells, and neutrophils (*p* < 0.01).

**Conclusion:**

A practical approach to investigating patients with persistent thrombocytosis based on clinical characteristics such as active malignancy, chronic inflammatory disease, splenectomy, and iron deficiency may assist in accurately identifying patients more likely to have secondary causes of thrombocytosis and reduce overinvestigation, particularly costly molecular testing.

## 1. Introduction

**Figure 1:**
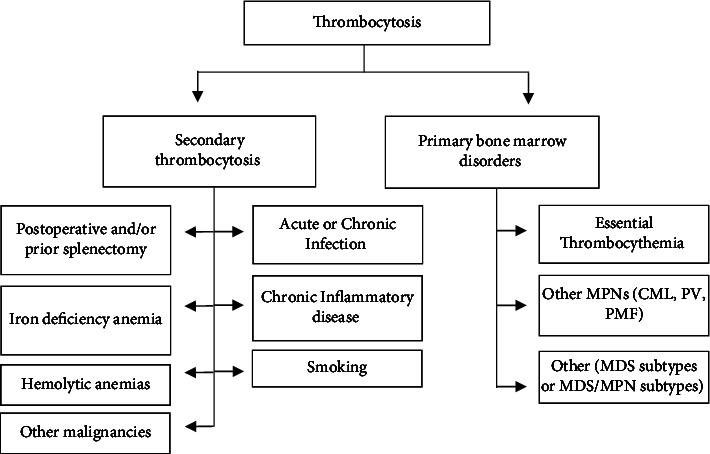
Differential diagnosis of thrombocytosis consisting of the most common causes of secondary thrombocytosis and primary bone marrow disorders. MPNs, myeloproliferative neoplasms; CML, chronic myeloid leukemia; PV, polycythemia vera; PMF, primary myelofibrosis; and MDS, myelodysplastic neoplasms.

Thrombocytosis defined as a platelet count ≥450 × 10^9^/L may be due to secondary causes, also known as reactive thrombocytosis, or primary bone marrow disorders such as essential thrombocythemia (ET). Differentiating between these entities can be challenging and is a common reason for referral to Hematology. Secondary causes of thrombocytosis include infection, chronic inflammatory disease, smoking, iron deficiency anemia, hemolytic anemias, postoperative state, such as postsplenectomy, as well as malignancy [1]. Primary causes of thrombocytosis include ET or other myeloproliferative neoplasms (MPNs) such as chronic myeloid leukemia (CML), polycythemia vera (PV), and primary myelofibrosis (PMF) among others (Figure 1).

The incidence of ET is estimated to be 0.2 to 2.7 per 100,000 population [2]. Clinically, patients with ET may present with asymptomatic thrombocytosis, constitutional symptoms (persistent unexplained fevers, drenching night sweats, and weight loss), splenomegaly, bleeding, or thrombosis [3, 4]. There is an increased risk of progression to secondary myelofibrosis and acute leukemia compared to the general population [5]. Advances in genomic technologies have expanded our knowledge of the mutational landscape of ET, PV, and MF [6]. ET is characterized by three canonical driver mutations: *JAK2*, *CALR*, and *MPL* [6]. *JAK2* mutations are the most common, found in 50–60% of patients, followed by *CALR* and *MPL* found in 15–30% and 1–4% of ET patients, respectively [7]. Patients who are “triple-negative” for these mutations account for the remaining 12% of ET cases [7]. These mutations are included in major diagnostic criteria for several MPNs by the 2022 World Health Organization (WHO) and the International Consensus Classification (ICC) [8, 9]. Genomic testing has become part of the standard of care for diagnosis of MPNs, which has been facilitated by the introduction of Next Generation Sequencing (NGS). NGS has become widely used in patients with suspected hematologic malignancies, raising concerns over high costs from indiscriminate utilization [10]. Given these concerns, there is growing interest in developing triage tools for investigation of myeloid malignancies [11], including prediction rules to help improve utilization of molecular testing in patients with suspected MPNs [12].

Most research to date has focused on detailed genomic characterization of patients with previously confirmed diagnosis of ET; by contrast, the utilization of molecular testing to investigate undifferentiated patients with thrombocytosis in real-world clinical environments remains underexplored. In this study, we characterize a cohort of patients referred for elevated platelet counts who underwent NGS testing and outline a practical diagnostic approach to thrombocytosis based on clinical characteristics aimed at improving utilization of molecular testing.

## 2. Methods

A retrospective chart review was conducted at the London Health Sciences Centre, an academic tertiary care centre in Southwestern Ontario, Canada. All adult patients (age ≥18 years) assessed between January 1, 2018 and May 31, 2021 for thrombocytosis (defined as platelet count ≥450 × 10^9^/L) who had NGS testing were included. NGS testing was performed using the Oncomine Myeloid Research Assay (Thermofisher Scientific, MA, USA). Demographic data collected included age and sex. Clinical history and factors collected included height, weight, smoking history, iron deficiency anemia, hemolytic anemia, recent hemorrhage within three months, acute or chronic infections, active malignancy, chronic inflammatory disorders (e.g., rheumatoid arthritis, inflammatory bowel disease, and sarcoidosis), splenectomy, recent surgery within three months, venous or arterial thrombosis, and thrombocytosis history. Laboratory data collected included complete blood count (CBC), reticulocyte count, serum erythropoietin (EPO), ferritin, transferrin saturation, erythrocyte sedimentation rate (ESR), C reactive protein (CRP), and myeloid NGS results.

Confirmed ET was defined as any patient meeting the WHO 2022 and ICC 2022 criteria for ET. Probable ET was defined as any patient meeting all WHO criteria but lacking a bone marrow biopsy. Probable and confirmed ET groups were compared to assess for significant differences between the two groups. The classification of secondary thrombocytosis was determined by the treating physician. Diagnoses were confirmed on chart review and any discrepancies were subsequently reviewed by consensus.

Laboratory and clinical variables were compared between patient in the combined confirmed and probable ET versus secondary thrombocytosis. We combined these two groups because, at our centre, hematologists did no routinely perform bone marrow biopsies on patients with probable ET, and the role of a bone marrow biopsy is primarily to exclude premyelofibrotic ET. To compare temporal patterns in platelet count between groups, change in platelet count was measured between the time of NGS testing and 6–12 months postdiagnosis. Patients who were on cytoreductive medications at the time were excluded from this temporal analysis. The prevalence of specific mutations associated with ET were identified, as well as the incidence of triple-negative ET. Student's *t*-test was used to calculate *p* values for baseline characteristics when comparing the two groups. Chi-square test was used to calculate *p* values for the clinical risk factors and NGS results. This study was approved by the Research Ethics Boards at Western University (120153) in accordance with the Declaration of Helsinki.

## 3. Results

A total of 441 patient charts were reviewed with 100 patients excluded due to meeting criteria for another MPN and 5 patients excluded for having an undetermined diagnosis. 336 patients (93 male and 243 female) were included for evaluation with mean age of 63 ± 18 years (Table 1). The ET group included 176 patients; the secondary thrombocytosis group included 160. To assess for differences within the ET group, we compared patients diagnosed with ET but who lacked a bone marrow biopsy (probable ET) to patients with ET confirmed by bone marrow biopsy (confirmed ET). The probable and confirmed ET groups were similar with respect to all clinical and laboratory features with the exception of platelet count, which was higher in the confirmed ET group compared to the probable ET group (924.13 ± 374.95 versus 761 ± 232.43, *p* < 0.0008; Supplemental Table 1). Both confirmed and probable ET groups, however, had significantly higher platelet count than the secondary thrombocytosis group, and were therefore combined for subsequent analyses.

The overall yield of NGS testing was 52.4% (176/336) with 92.05% (162/176) of patients in the ET group having one of three canonical driver mutations: *JAK2* (52.0%), *CALR* (29.0%), and *MPL* (8.3%). Of these patients, 14.2% (25/176) had additional mutations alongside the known driver mutation found on their NGS panel. Patients in the secondary thrombocytosis group were generally younger than the confirmed and probable ET groups (56 vs. 69 years, *p* < 0.0001) and had higher body mass index (30 vs. 27 kg/m^2^, *p* < 0.0001). Clinical variables also differed between the ET group and the secondary thrombocytosis group. A previous history of arterial thrombosis was predictive of probable ET (*p* < 0.01), while other clinical factors such as chronic inflammatory disease (*p* < 0.01), previous splenectomy (*p*=0.05), and iron deficiency anemia (*p*=0.01) were associated with a diagnosis of secondary thrombocytosis. The incidence of smoking differed between the two groups 68 (53%) in the ET group versus 65 (37%) in the secondary thrombocytosis group, but did not reach statistical significance. Furthermore, there were significant differences in hemoglobin (122.92 ± 21.85 vs. 131.84 ± 17.80, *p* < 0.0001), MCV (87.72 ± 7.75 vs. 90.41 ± 9.236, *p*=0.0049), MPV (9.18 ± 0.94 vs. 9.57 ± 1.06), *p* < 0.0001), platelet count (562 ± 140.75 vs. 802.87 ± 267.59, *p* < 0.0001), change in platelet counts (87.25 ± 222.71 vs. −226.44 ± 350, *p* < 0.0001), and neutrophil counts (8.15 ± 7.25 vs. 6.46 ± 3.39, *p* < 0.01) between the secondary thrombocytosis group and the ET group (Table 1, *p* < 0.01). The mutational characteristics of the ET group were also investigated between patients who had single driver mutations of *JAK2*, *CALR* type 1 and 2, and/or *MPL* (Table 2).

## 4. Discussion

Essential thrombocythemia can present with isolated thrombocytosis with or without associated symptoms. Distinguishing between ET and secondary thrombocytosis can be clinically challenging. Our study suggests that several clinical variables, such as a history of thrombosis, chronic inflammatory disease, splenectomy, and iron deficiency anemia, as well as laboratory parameters, including CBC and differential white blood cell counts, can be useful in helping differentiate between these two conditions.

Findings from our study could assist clinicians in limiting overinvestigation of this common referral population. Improved access to molecular testing has facilitated the diagnosis of ET at earlier stages [4] but has also led to an increase in utilization of molecular testing as an upfront test often simultaneously ordered in the work up for patients referred for thrombocytosis. Although molecular testing has a relatively high specificity and sensitivity for MPNs, it is a costly tool that may be avoided in patients who more likely have secondary thrombocytosis. Although these clinical factors cannot entirely exclude ET, findings from our study suggest that many patients with clear secondary causes could be observed rather than doing upfront molecular diagnostics.

**Figure 2:**
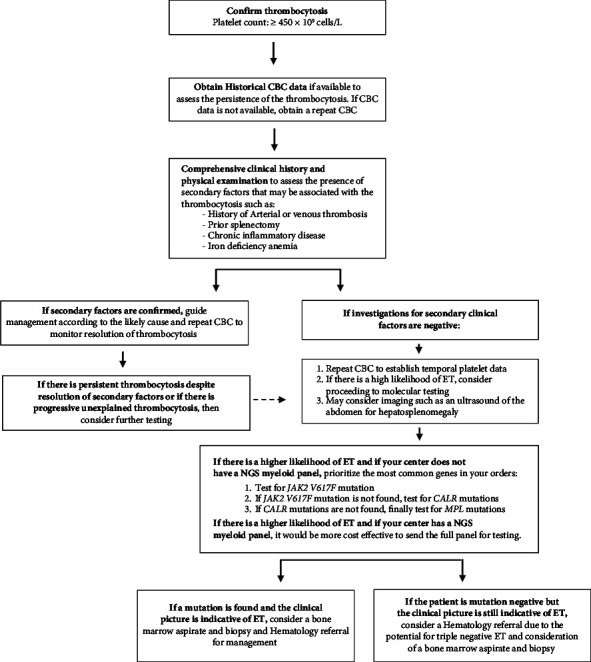
Approach to the diagnostic workup of patients presenting with thrombocytosis. The proposed approach has not been validated and is not meant to replace clinical judgment. CBC, complete blood count; ET, essential thrombocythemia; *JAK2*, Janus Kinase 2 gene; *CALR*, Calreticulin gene; and *MPL*, thrombopoietin receptor protein gene.

We suggest a rational diagnostic approach (Figure 2) beginning with a CBC to confirm presence of persistent thrombocytosis, defined as a platelet count of ≥450 × 10^9^ cells/L, and to assess for findings suggestive of other MPNs, such as erythrocytosis in the case of polycythemia vera, or granulocytosis in the case of CML [13, 14]. In patients with isolated thrombocytosis, the WHO 2022 and ICC 2022 criteria for ET include persistent thrombocytosis, but a specific length of time is not clearly defined. Historic CBC data may help establish the chronicity of thrombocytosis. Although in this cohort the baseline platelet count was significantly higher in the ET group when compared to the secondary thrombocytosis group (802.9 ± 267.6 versus 562.0 ± 140.8, *p* < 0.0001), the considerable overlap in range between the 2 groups makes platelet count alone a poor discriminator despite previous literature associating primary with higher platelet counts [15].

Once isolated and persistent thrombocytosis is confirmed, a comprehensive work up for secondary causes should include history of smoking, use of medications, recent malignancy, chronic inflammatory disease, recent surgery such as splenectomy, recent infections, iron deficiency anemia, and hemolytic anemia [16]. If secondary factors are discovered and can be treated, management can be guided as appropriate and monitoring of the resolution of thrombocytosis is suggested. Alternatively, if secondary factors are not identified or there is history of thrombotic events, such as arterial thrombosis, ET should be suspected, and molecular testing would be indicated. If a myeloid NGS panel is available at a centre, it may be more cost effective and comprehensive to order the full myeloid NGS panel as opposed to individual PCR assays for *JAK2*, *CALR* type 1 and 2, and/or *MPL* [10, 17]. However, if an NGS panel is not available, molecular testing should prioritize the most common mutation found in patients with ET.

Based on the findings from our study, we propose a stepwise diagnostic approach to patients referred for elevated platelet counts that begins with exclusion of common causes that are highly predictive of secondary thrombocytosis or ET (Figure 2). Specifically, we identified arterial thrombosis as a predictor for ET whereas iron deficiency anemia, chronic inflammatory disease, active malignancy, splenectomy, and smoking are often positive predictors of secondary thrombocytosis. These clinical correlates can assist in deciding whether upfront molecular testing is required. If the work up is negative for clinical factors that points to secondary thrombocytosis, then molecular testing may be appropriate. Although patients with ET were generally older (68 vs. 56 years, *p* < 0.0001), had lower BMI (27 vs. 30 kg/m^2^, *p* < 0.0001), and had a higher platelet count than patients with secondary thrombocytosis (802.9 ± 267.6 versus 562.0 ± 140.8, *p* < 0.0001), these parameters cannot independently exclude ET but may be useful as part of multivariable prediction rule to predict ET alongside WBC and other CBC parameters. Additionally, elevated BMI has been associated with mild neutrophilia attributed to proinflammatory cytokines released by adipose cells [15]. Previous studies have linked indicators of obesity, such as abdominal height, to increase platelet counts as well [18]. These variables may further assist in the creation of multivariable prediction tool in a similar approach as the JAK2 positive PV prediction tool [1].

The strength of this diagnostic approach of thrombocytosis is its simplicity and reliance on readily available clinical characteristics based on history and limited number laboratory test including CBC and ferritin. Additional laboratory markers of inflammation such as CRP or ESR may also be useful but were not available for all patients in our study. Additional limitations include the retrospective nature of this study. As a tertiary care centre database, referred patients may have been less likely to have obvious secondary causes of thrombocytosis already excluded in primary care. This may limit the ability to generalize our findings to primary care practices, but we suspect the algorithm may have even greater utility in primary care where the prevalence of secondary thrombocytosis is higher. Although a bone marrow biopsy is part of the WHO 2022 and ICC 2022 diagnostic criteria for ET [8, 9], many patients in our study with an ET diagnosis lacked a bone marrow biopsy. There is some debate regarding whether a bone marrow biopsy is clinically necessary in the diagnosis of ET as some studies highlight the value of the test allowing for more sensitive differentiation between ET and reactive causes [19, 20]. Moreover, some have argued that the value of biopsy is to help distinguish ET from other chronic MPNs, most commonly prefibrotic primary myelofibrosis or PV [21]. We acknowledge that probable ET patients in our cohort who did not have biopsy may have included early cases of myelofibrosis, but this would not have impacted the initial diagnostic approach. The discovery of mutational landscapes and driver mutations, such as the *JAK2* mutation, have assisted in the diagnosis of ET [22]. Our proposed approach is intended to provide general guidance to consulting physicians in the initial investigation of patients referred for thrombocytosis. It is not intended as a substitute for clinical judgment. Awareness and exclusion of common, secondary causes of thrombocytosis could help avoid unnecessary, invasive testing such as bone marrow examination, and improve utilization of costly molecular testing. Further research, however, is required to provide definitive guidance on indications for molecular testing, including strategies to efficiently triage patients with high pretest probability of ET.

## 5. Conclusion

This diagnostic approach is proposed as a practical guide for clinicians based on current available evidence, including findings from our cohort. The diagnostic performance and cost effectiveness of this approach, however, remains to be formally evaluated. The commonly available clinical and laboratory parameters may be useful for the development of multivariable model to predict ET and guide testing in the future.

## Figures and Tables

**Table 1:** Baseline characteristics, clinical risk factors for thrombocytosis, and myeloid NGS result.

Characteristics of cohort	Secondary thrombocytosis *N* = 160	Essential thrombocythemia (ET) *N* = 176	*p* value
Age (years; mean ± SD)^*∗*^	56 ± 17	69 ± 15	<0.0001
Gender: male (%), female (%)	28 (18.5), 132 (82.5)	65 (36.9), 111 (63.1)	
BMI (kg/m^2^; mean ± SD)	30 ± 8.3	27 ± 5.4	<0.0003
WBC (×10^9^ cells/L; mean ± SD)	13.2 ± 11.9	9.4 ± 4.1	<0.0001
Hemoglobin (g/L; mean ± SD)	122.9 ± 21.9	131.8 ± 17.8	<0.0001
MCV (fL; mean ± SD)	87.7 ± 7.8	90.41 ± 9.236^a^	0.0049
RDW (%; mean ± SD), (*N* = 145, 152)	15.3 ± 2.5	15.5 ± 2.5	0.3710
MPV (fL; mean ± SD)	9.2 ± 0.9	9.6 ± 1.1	<0.0004
Platelet count (×10^9^ cells/L; mean ± SD)	562 ± 141	803 ± 284	<0.0001
Change in platelet count (×10^9^ cells/L; mean ± SD)^*∗∗*^	87 ± 223	−226 ± 350	<0.0001
ANC (×10^9^ cells/*µ*L; mean ± SD)	8.2 ± 7.3	6.5 ± 3.4	0.0058
Ferritin (*μ*g/L; mean ± SD)	172 ± 320	148 ± 154	0.4522

*Clinical risk factors for thrombocytosis* ^*∗∗∗*^			
Smoking, *n* (%)	68 (53)	65 (37)	0.2113
Arterial, venous thrombosis, *n* (%)	16 (10), 5 (3)	39 (22), 9 (5)	0.0026, 0.3623
Prior splenectomy, *n* (%)	10 (63)	3 (1.7)	0.0310
Active malignancy, *n* (%)	11 (6.9)	4 (2.3)	0.0413
Chronic inflammatory disease, *n* (%)	19 (11.9)	7 (4.0)	0.0068
Iron deficiency anemia, *n* (%)	25 (15.6)	12 (6.8)	0.0100

*Myeloid NGS results*			
*JAK2 V617F*; *CALR Type 1* and *Type 2*; *MPL*	NA	95 (54), 31 (18), 20 (11), 16 (9), ^*∗∗∗∗*^	
Triple negative, *n* (%)	NA	9 (5)	
Other myeloid mutations, *n* (%)^*∗∗∗∗*^	15 (9.7)	29 (16.5)	0.0539
	*ASXL1*, 3 (1.9)	*TET2*, 7 (4.0)	
	*DNMT3A*, 7 (4.4)	*ASXL1*, 5 (2.8)	
	Other 5 (3.1)	*SF3B1*, 5 (2.8)	
		Other 12 (6.8)	

^*∗*^Age was determined at the time of NGS testing. ^*∗∗*^Change in platelet count included a total of 75 patients (23 in the ET group and 52 in the secondary thrombocytosis group) with evaluable data and excludes any patients on cytoreductive therapy. ^*∗∗∗*^Patients may have multiple secondary causes. ^*∗∗∗∗*^Some patients may have multiple mutations (3 patients in the secondary thrombocytosis had multiple mutations and 25 patients in the ET group had a second mutation alongside their driver mutation). ^a^MCV was calculated on the 159 patients not on any cytoreductive therapy at the time of NGS testing (17 patients were on hydroxyurea). BMI, body mass index; WBC, white blood cells; MCV, mean corpuscular volume; RDW, red cell distribution width; MPV, mean platelet volume; ANC, neutrophil count; *JAK2*, Janus Kinase 2 gene; *CALR*, Calreticulin gene; *MPL*, thrombopoietin receptor protein gene.

**Table 2:** Baseline characteristics and clinical risk factors for thrombocytosis categorized based on mutational landscape of ET patients with a single driver mutation.

	*JAK2* (*N* = 78)	*CALR* ^*∗*^ (*N* = 46)	*MPL* (*N* = 13)
*Demographic characteristics*			
Age (years; mean ± SD)	72 ± 15	62 ± 15	72 ± 11
Gender (female *n* (%), male *n* (%))	51 (65), 27 (35)	24 (51), 22 (47)	10 (77), 3 (23)
BMI (kg/m^2^; mean ± SD)	26.0 ± 4.8	29.3 ± 6.1	28.1 ± 6.5

*CBC characteristics*			
WBC (×10^9^ cells/L; mean ± SD)	10.0 ± 4.2	8.0 ± 3.3	6.9 ± 3.1
Hemoglobin (g/L; mean ± SD)	135 ± 16.4	132 ± 14.3	125 ± 17.4
MCV (fL; mean ± SD)	93 ± 10.0	95 ± 9.7	103 ± 12.6
RDW (%; mean ± SD)	15.3 ± 2.6	14.9 ± 1.9	14.4 ± 2.1
MPV (fL; mean ± SD)	9.8 ± 1.0	9.2 ± 1.0	9.5 ± 1.2
Platelet count (×10^9^ cells/L; mean ± SD)	777 ± 290	863 ± 264	619 ± 205
ANC (×10^9^ cells/*µ*L; mean ± SD)	6.9 ± 3.6	5.2 ± 2.5	4.8 ± 2.0
Ferritin (*μ*g/L; mean ± SD)	115 ± 87	200 ± 186	215 ± 189

*Clinical characteristics*			
Smoking, *n* (*N*, %)	30 (64, 47)	13 (37, 35)	4 (6, 67)
Arterial, venous thrombosis, *n* (%)	22 (28), 6 (8)	9 (19), 0 (0)	1 (8), 0 (0)
Prior splenectomy, *n* (%)	1 (1)	1 (2)	0 (0)
Active malignancy, *n* (%)	3 (4)	0 (0)	0 (0)
Chronic inflammatory disease, *n* (%)	3 (4)	2 (4)	1 (8)
Iron deficiency anemia, *n* (%)	5 (6)	4 (8)	1 (8)

^*∗*^ *CALR* mutation category includes type 1 and type 2. BMI, body mass index; WBC, white blood cells; MCV, mean corpuscular volume; RDW, red cell distribution width; MPV, mean platelet volume; ANC, neutrophil count; *JAK2*, Janus Kinase 2 gene; *CALR*, Calreticulin gene; *MPL*, thrombopoietin receptor protein gene; *N*, sample size; *n*, number of patients.

## Data Availability

The data used in this study are available upon request to the corresponding author.

## Supplementary Materials

Supplementary Materials Supplemental Table 1: statistical comparison of the confirmed ET and probable ET groups. Summary of Supplemental Table 1: this table provides a comparison of clinical and laboratory variables between the confirmed ET and probable ET groups.
