# Supplementary material for: An Approach to the Investigation of Thrombocytosis: Differentiating between Essential Thrombocythemia and Secondary Thrombocytosis
Source: Adv Hematol. 2024 Feb 12;2024:3056216. doi: 10.1155/2024/3056216 (PMC10876298; doi:10.1155/2024/3056216)
Supplement: Supplementary Materials — Supplemental Table 1: statistical comparison of the confirmed ET and probable ET groups. Summary of Supplemental Table 1: this table provides a comparison of clinical and laboratory variables between the confirmed ET and probable ET groups. [file 3056216.f1.docx]

**Supplemental Table**

**Supplemental Table 1.** Statistical comparison of the confirmed ET and probable ET groups.

| **Characteristics of Cohort** | **Confirmed ET**  **N = 45** | **Probable ET p-value**  **N = 131** |
| --- | --- | --- |
| Age (years; mean+/-SD)  Gender: male (%), female (%) | 67 +/- 15  16 (36), 29 (64) | 70 +/- 15 0.2487  49 (37), 82 (63) |
| BMI (kg/m^2^; mean +/- SD) | 27.6 +/- 5.0 | 27.2 +/- 5.6 0.6565 |
| WBC (x 10^9^ cells /L; mean +/- SD) | 10.3 +/- 4.7 | 9.1 +/- 3.8 0.0985 |
| Hemoglobin (g/L; mean +/- SD) | 127 +/- 23.2 | 133 +/- 15.3 0.0548 |
| MCV (fL; mean +/- SD) | 95 +/- 13.5 | 94 +/- 9.7 0.0500 |
| **RDW (%; mean +/- SD**), | 15.2 +/- 2.8 | 15.6 +/- 2.4 0.4203 |
| MPV (fL; mean +/- SD) | 9.4 +/- 1.2 | 9.6 +/- 1.0 0.1411 |
| Platelet Count (x 10^9^ cells /L; mean +/- SD)) | 924 +/- 375 | 761 +/- 232 0.0008 |
| Change in platelet count (x 10^9^ cells /L; mean +/- SD) | -234 +/- 507 | -217 +/- 267 0.7864 |
| ANC (x 10^9^ cells/µL; mean +/- SD) | 7.3 +/- 3.8 | 6.2 +/- 5.4 0.2288 |
| Ferritin (μg/L; mean +/- SD) | 170 +/- 160 | 141 +/- 153 0.2754 |
| **Clinical Risk Factors for thrombocytosis*** |  |  |
| Smoking, *n (*%, N) | 17 (N=37) | 48 (N=107) 0.1024 |
| Arterial, Venous thrombosis, *n* (%) | 9 (20), 3 (7) | 30 (67), 6 (5) 0.2042, 0.5836 |
| Prior splenectomy, *n* (%) | 1 (11) | 2 (2) 0.7558 |
| Active malignancy, *n* (%) | 0 (0) | 4 (3) 0.2357 |
| Chronic inflammatory disease, *n* (%) | 2 (4) | 5 (4) 0.8525 |
| Iron deficiency anemia, *n* (%) | 3 (7) | 9 (7) 0.9627 |
| **Myeloid NGS results **** |  |  |
| *JAK2 V617F; CALR Type 1, Type 2; MPL* | 19 (42), 7 (16), 5 (11), 4 (9) | 76 (58), 24 (18), 15 (11), 12 (9) |
| Triple Negative, n (%) | 10 (22) | 4 (3) |
| Other Myeloid Mutations, n (%)  Multiple mutations  associated with driver mutation | 6 (13)  *ASXL1*, 2 (4)  *U2AF1*, 2 (4)  *TP53*, 1 (2)  *IDH2*, 1 (2)  4 (9) | 23 (18)  *TET2*, 7 (5)  *SF3B1*, 5 (4)  *ASXL1*, 4 (3)  Other 7 (5)  21 (16) |

BMI – Body mass index; WBC – White blood cells; MCV - Mean corpuscular volume; RDW – Red cell distribution width; MPV – Mean platelet volume; ANC – Neutrophil count; *JAK2* – Janus Kinase 2 gene; *CALR* – Calreticulin gene; *MPL* – thrombopoietin receptor protein gene

* Patients may have multiple secondary causes

** Some patients may have multiple mutations
